# Progress in the total synthesis of resin glycosides

**DOI:** 10.3389/fchem.2022.1036954

**Published:** 2022-11-11

**Authors:** Wenli Wang, Yuxin Li, Ye He, Xing Jiang, Ying Yi, Xihan Zhang, Shiyu Zhang, Guangtong Chen, Min Yang, Jia-Lie Luo, Boyi Fan

**Affiliations:** ^1^ School of Pharmacy, Nantong University, Nantong, China; ^2^ Jinghua Pharmaceutical Group Co., Ltd., Nantong, China

**Keywords:** resin glycosides, total synthesis, glycolipids, macrolactonization, glycosylation

## Abstract

Resin glycosides, mainly distributed in plants of the family Convolvulaceae, are a class of novel and complex glycolipids. Their structural complexity and significant biological activities have received much attention from synthetic chemists, and a number of interesting resin glycosides have been synthesized. The synthesized resin glycosides and their analogues not only helped in structural verification, structural modification, and further biological activity exploration but also provided enlightenment for the synthesis of glycoside compounds. Herein, the present review summarizes the application of various efforts toward the synthesis of resin glycosides in the last decade.

## Introduction

Natural products have significantly contributed to the development of new drugs for human disorders (Newman and Cragg, 2020). Resin glycosides are a class of novel and complex natural glycolipids mainly discovered in plants of the family Convolvulaceae. Because of the diversified structures and various bioactivities, resin glycosides have attracted plenty of interests from organic chemists and pharmacologists (Pereda-Miranda et al., 2010; Fan et al., 2022). Up to now, hundreds of resin glycosides and their derivatives have been identified and reported, some of which exhibited significant biological activities, such as cytotoxic (Fan et al., 2014; Ono et al., 2019), anti-viral (Ono et al., 2014), anticonvulsant (Castro-García et al., 2018), anti-tumor migration (Chen et al., 2017), sedative (León-Rivera et al., 2014), vasorelaxant (León-Rivera et al., 2014), and α-glucosidase inhibitory effects (Pan et al., 2015), as well as multidrug-resistant reversal effects on both microbial pathogens and mammalian cancer cells (Figueroa-González et al., 2012; Wang et al., 2018; Lira-Ricárdez and Pereda-Miranda. 2020), indicating their potential as lead compounds for drug discovery. However, not enough in-depth biological exploitation has been taken place. Possible reasons could be the difficulties on the isolation and purification.

The structures of resin glycosides consist of three ingredients: oligosaccharide chain, long-chain fatty acid aglycone, and modified acyl groups (Pereda-Miranda et al., 2010; Fan et al., 2022). The sugar units of oligosaccharides mostly range from three to seven, with D-glucose (Glc), L-rhamnose (Rha), D-fucose (Fuc), D-quinovose (Qui), D-xylose (Xyl), and D-galactose (Gal) as the monosaccharide unit. The aglycones are usually monohydroxy to trihydroxy C14–C17 long-chain fatty acids, which often link through an intramolecular esterification with the oligosaccharide chain to form a macrolactone ring. The oligosaccharide residues are also modified with some fatty acids (Fan et al., 2022). Due to the variation in the oligosaccharide units and sequence, aglycone, macrolactone site, acylated residues, and the formation of the dimers, the structural complexity of resin glycosides has received much attention from synthetic chemists.

**FIGURE 1:**
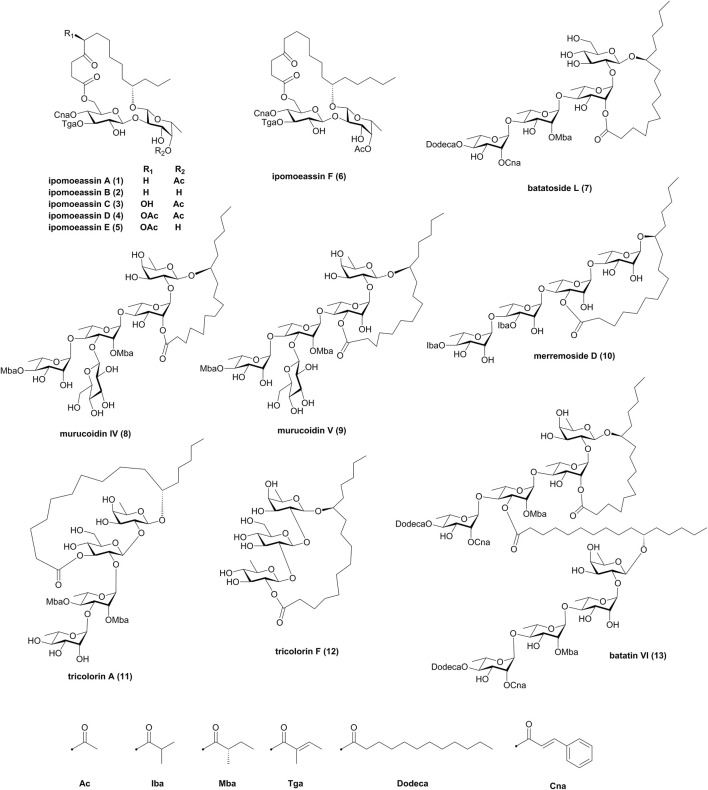
Structures of resin glycosides **1**–**13**. Iba, isobutyryl; Mba, 2*S*-methylbutyryl; Tga, tiglyl; Dodeca, n-dodecanoyl; Cna, *trans*-cinnamyl.

The first total synthesis of resin glycosides was published in the 1990s (Lu et al., 1997), and then many resin glycosides have been synthesized successfully. The construction of the macrolide structures and oligosaccharide chains presented a major challenge to develop methodologies for their synthesis. In 2010, Pereda-Miranda and coworkers reviewed the state of resin glycoside synthesis to early 2009 (Pereda-Miranda et al., 2010). Since then, more total synthesis research studies on resin glycosides have been achieved and published. Therefore, to obtain a comprehensive perspective, the present review provides a systematic summary of synthetic strategies for the total synthesis of resin glycosides over the period from 2009 to the end of 2021. Several electronic databases, including the Web of Science, PubMed, Scopus, and Google Scholar, were searched using the keyword “resin glycoside” paired with “synthesis” or “total synthesis.” A total of 13 resin glycosides, as shown in Figure 1, have been synthesized during this period, including ipomoeassins A–F (**1**–**6**), batatoside L (**7**), murucoidins IV (**8**) and V (**9**), merremoside D (**10**), tricolorins A (**11**) and F (**12**), and batatin VI (**13**).

## Total synthesis of ipomoeassins A–F (1–6)

Ipomoeassins A–F (**1–6**) are a kind of disaccharide resin glycosides isolated from the leaves of *Ipomoea squamosa* in the Suriname rainforest (Cao et al., 2005; Cao et al., 2007). Among them, ipomoeassins D (**4**) and F (**6**) showed exceptionally potent low cytotoxicity to single-digit nanomolar IC_50_ values against several cancer cell lines. Therefore, these natural glycoconjugates quickly inspired synthetic chemists to tackle their total synthesis.

### Approach by Fürstner’s group

Fürstner’s group has reported the first total synthesis of ipomoeassins B (**2**) and E (**5**) based on a ring-closing olefin metathesis (RCM) reaction for the formation of the macrocycle at the C8–C9 bond of the aglycone (Fürstner and Nagano. 2007). They also provided access to all naturally occurring ipomoeassins and a few analogues, together with the SAR of these compounds (Nagano et al., 2009). Although the total synthesis of ipomoeassins E (**5**) by Fürstner’s group has been reviewed by Pereda-Miranda et al. (2010), here we give a more comprehensive review on the Fürstner’s work.

**FIGURE 2:**
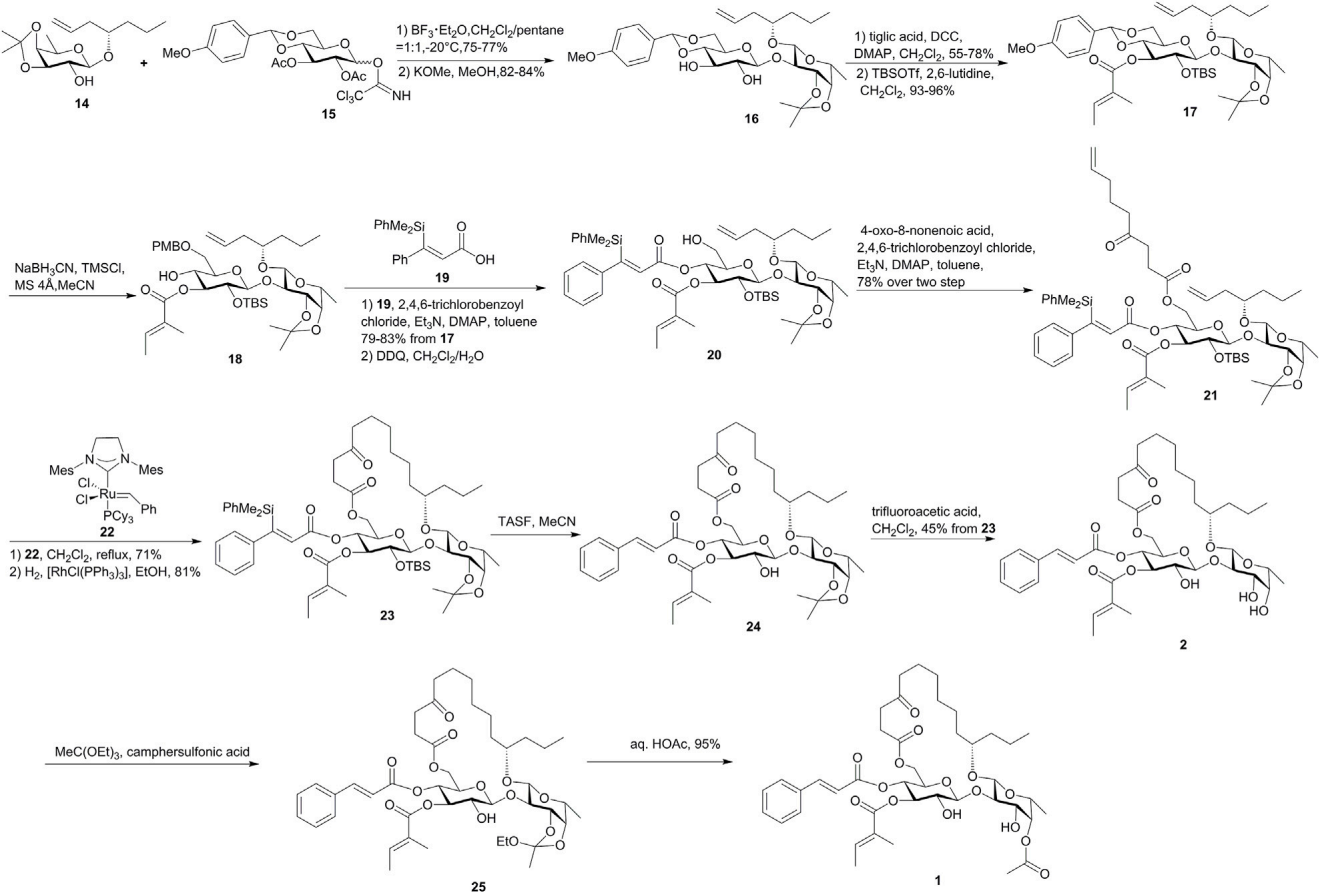
Fürstner’s approach toward the synthesis of ipomoeassins A–F (**1**–**6**). TASF, tris(dimethylamino)sulfonium difluorotrimethylsilicate.

**FIGURE 3:**
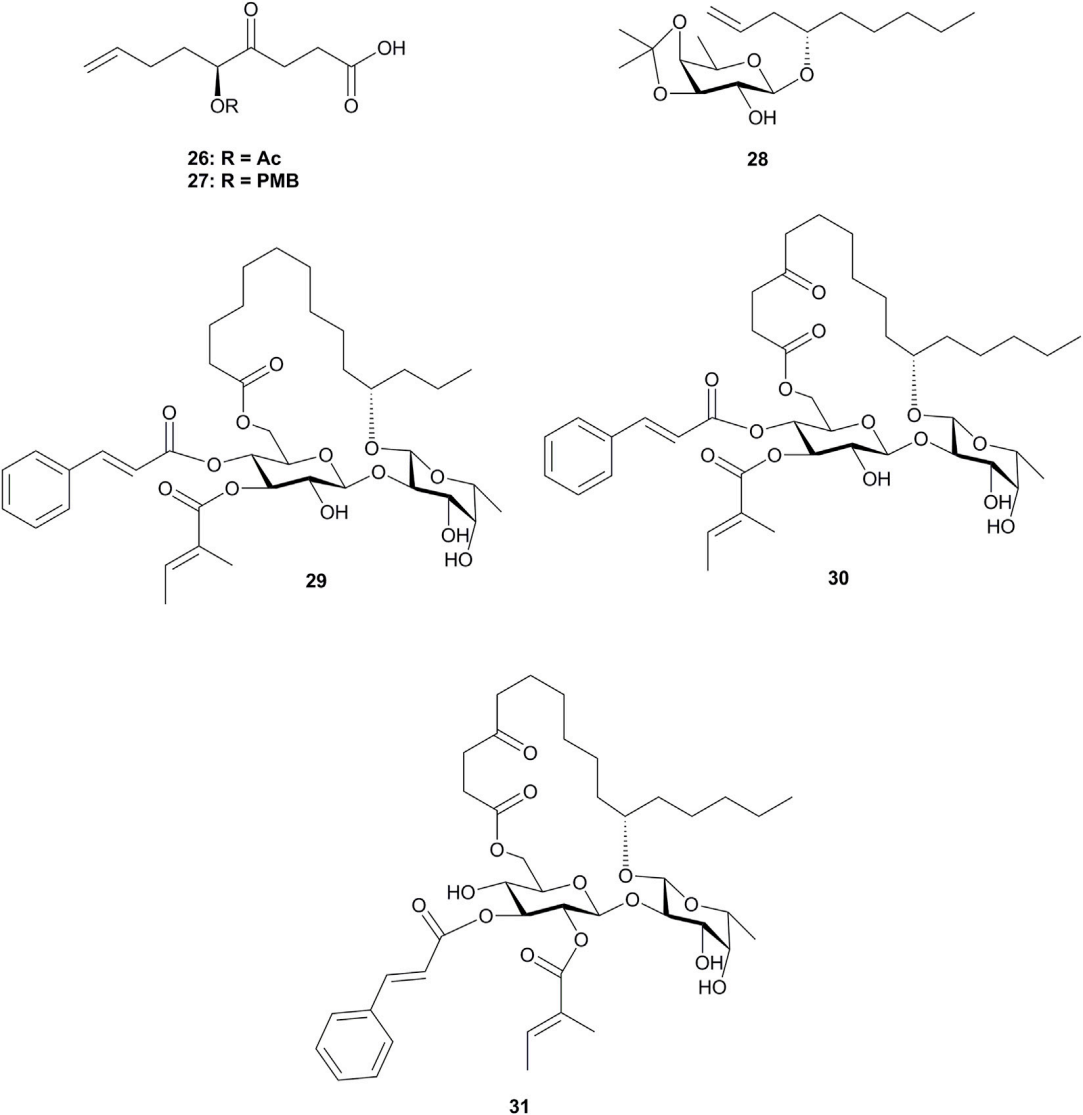
Structures of **26**–**31**.

As shown in Figure 2, the route by Fürstner’s group relied on the use of compound **19** as a new cinnamic acid surrogate, which was hydrogenation-resistant over Wilkinson’s catalyst and allowed the unsaturated acids to be attached at an early phase of the synthesis. The key disaccharide **16** was assembled by a BF_3_·Et_2_O-mediated glycosidation method and was esterified with tiglic acid at the more nucleophilic O-3 site of *β*-glucoside to form **17**. Then, the reductive opening of the substituted benzylidene acetal using NaBH_3_CN in combination with TMSCl furnished a mixture of 6-OPMB ether (**18**) and 4-OPMB ether in up to 84% combined yield with a 4:1 isomeric ratio. Alcohol **18** was esterified with **19** under Yamaguchi conditions, which was then subjected to oxidative PMB cleavage followed by the attachment of the 4-oxo-8-nonenoic acid ester segment. The resulting diene **21** was treated with catalytic amounts of ruthenium alkylidene complex **22** for RCM macrolactonization and then was hydrogenated with the aid of [RhCl(PPh_3_)_3_] to afford macrolactone **23**. After cleavage of the *C*-silyl and *O*-silyl groups and deprotection of isopropylidene acetal, ipomoeassin B (**2**) was obtained. Ipomoeassin A (**1**) was converted from ipomoeassin B (**2**) by treating with MeC(OEt)_3_ in the presence of camphersulfonic acid, followed by an HOAc-induced rearrangement. The synthesis of ipomoeassins D (**4**) and E (**5**) was very similar with ipomoeassins A (**1**) and B (**2**), respectively, with the only difference on the use of a synthetic chiral acid **26** (Figure 3) for the esterification of the primary alcohol in **20**. The PMB-protected acid **27** (Figure 3) was used to form diene in the synthesis of ipomoeassin C (**3**), which was cleaved with DDQ to form the hydroxyl group in the C-5 position of the aglycone moiety. Ipomoeassin F (**6**) differs from ipomoeassin A (**1**) only by the presence of two additional methylene units in the tail of aglycone, and no synthetic strategy was changed for the ipomoeassin F (**6**), except for the substitute of heptenol with nonenol to form the building block **28** (Figure 3) instead of **14**.

Apart from ipomoeassins A–F (**1**–**6**), three analogues **29**–**31** (Figure 3) were also synthesized by Fürstner’s group. By comparing the cytotoxic activity of these compounds, it was observed that the acetylation of the axial 4-OH group of fucopyranose and hydroxylation of the C-5 position in the lipophilic tether are detrimental. In addition, the ketone in the tether, the exact location of the two unsaturated esters, and the lipophilicity/hydrophilicity balance also showed strong influences on the bioactivity. A cell cycle analysis showed that those ipomoeassins could cause G_0_/G_1_ arrest in L-929 cells, followed by the apoptotic induction (Nagano et al., 2009).

### Approach by Postema’s group

The synthetic approach for ipomoeassin F (**6**) proposed by Postema’s group also used RCM as the macrolactonization method and differed with that of Fürstner by the introduction of the unsaturated acid in the late stage (Postema et al., 2009, Figure 4).

**FIGURE 4:**
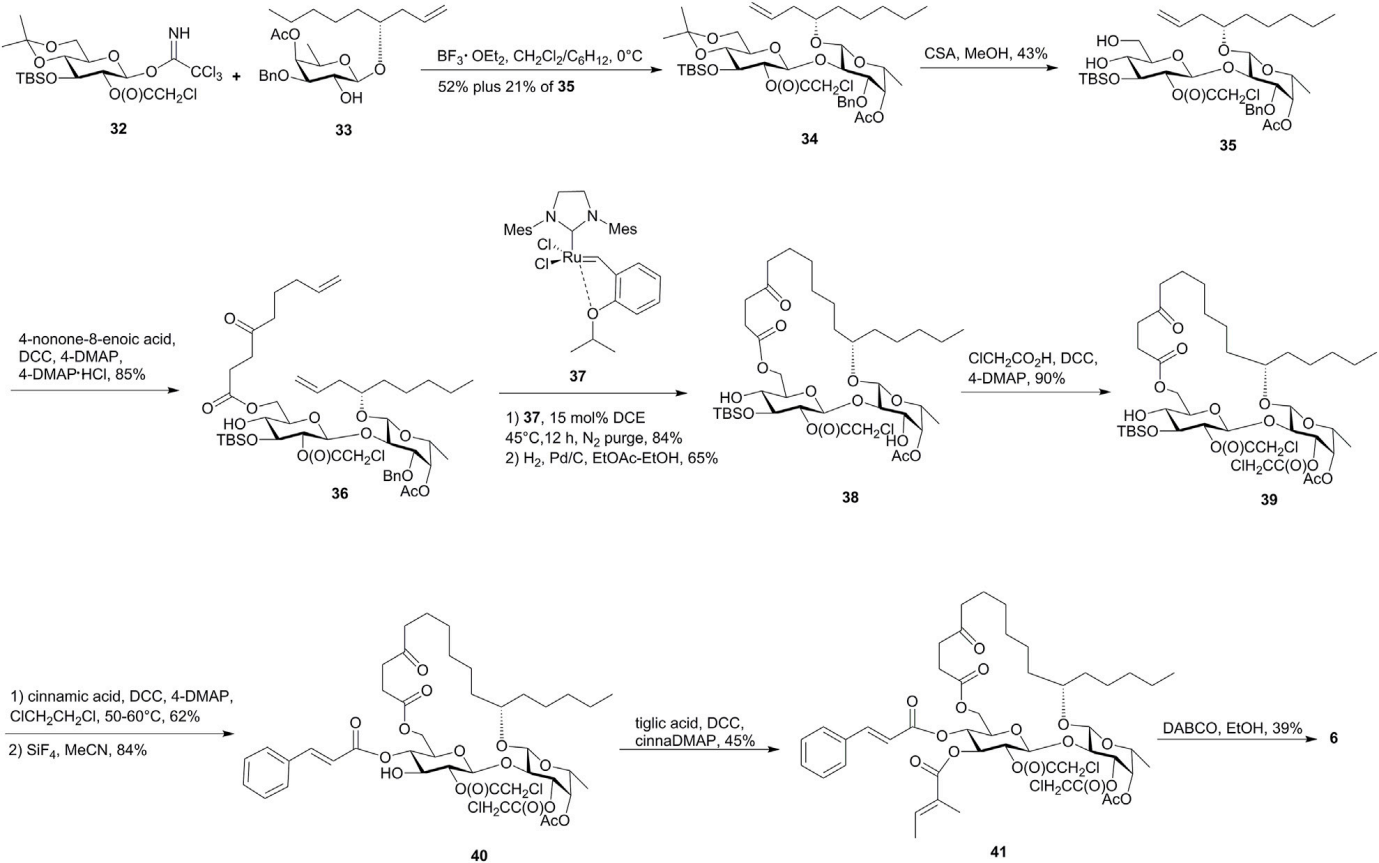
Postema’s approach toward the synthesis of ipomoeassin F (**6**). CSA, camphor-10-sulfonic acid.

In this approach, the obtained diene (**36**) cleanly cyclized to the desired 20-membered ring on reaction with ruthenium carbine (**37**), which was directly subjected to hydrogenation and cleavage of the *O*-3 benzyl group of fucopyranose to deliver **38**. The subsequent selective chloroacetylation on the *O*-3 hydroxyl group of fucopyranose afforded **39**. After the installment of the cinnamoyl group at the *O*-4 hydroxyl of the glucopyranose, the TBS group was effectively removed and following tigloylation gave the fully blocked precursor **41**. Finally, the removal of *R*-chloroacetates by employing an excess of DABCO furnished ipomoeassin F (**6**) in 39% yield after purification by flash chromatography and HPLC.

### Approaches by Shi’s group

Two strategies for the construction of ipomoeassin F (**6**) were described by Shi and colleagues and were both based on the RCM in constructing ring structures, with the difference on the introduction stage of the tiglate moiety (Zong et al., 2015; Zong et al., 2017b).

**FIGURE 5:**
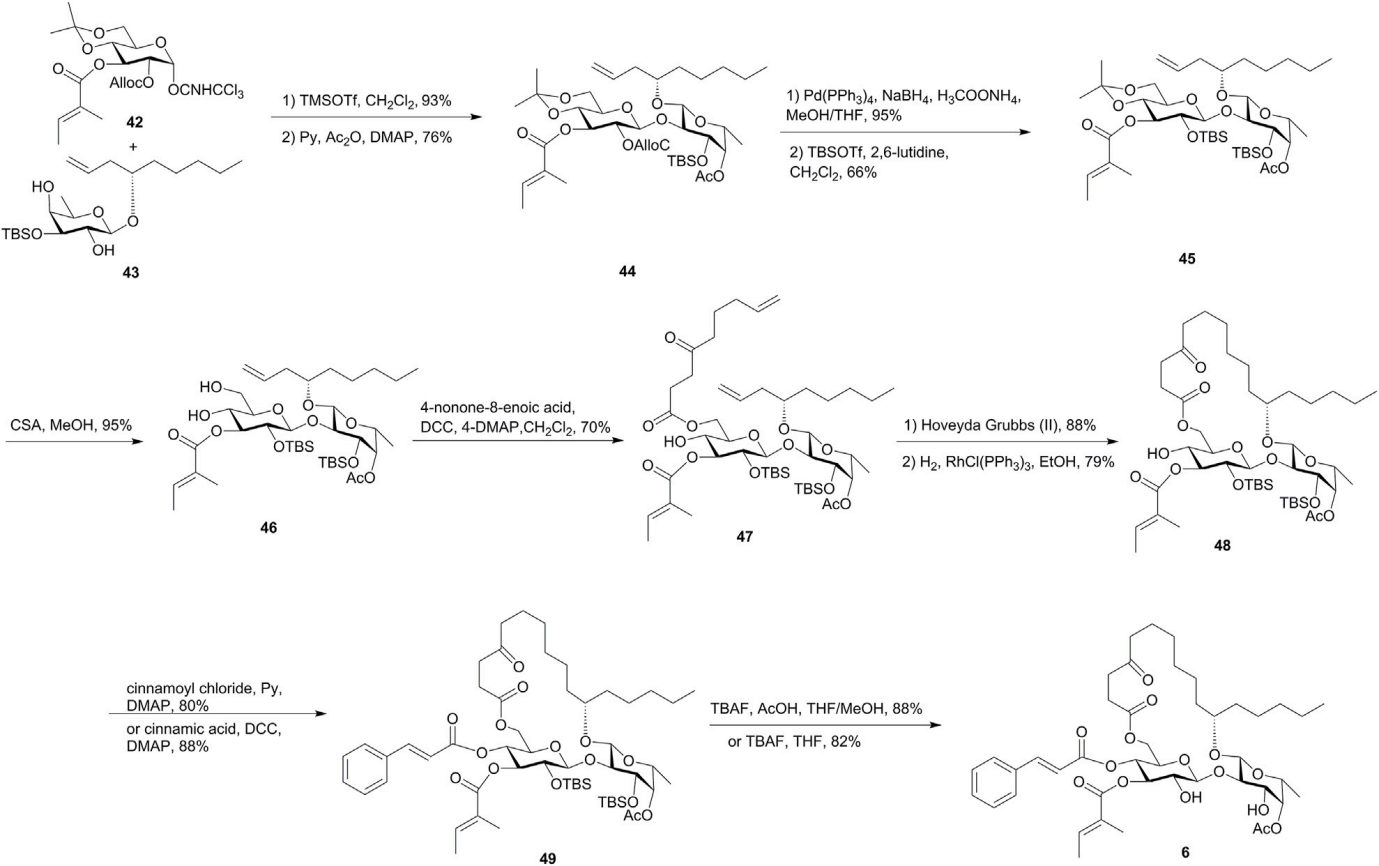
First Shi’s approach toward the synthesis of ipomoeassin F (**6**). Alloc, allyloxycarbonyl; CSA, camphor-10-sulfonic acid.

In the first strategy shown in Figure 5, the tiglate was introduced into the glucosyl donor **42** in an early stage, and the fucosyl acceptor **43** was designed with minimal protection steps from the fucosyl donor and chiral alcohol. The regioselective Schmidt glycosylation of **43** and **42**, followed by the acetylation of the remaining hydroxyl group, gave disaccharide **44** in 71% yield. The resulting alcohol obtained by the removal of the Alloc group was treated with TBSOTf and 2,6-lutidine to afford the silyl ether **45** as the major product (66%). The removal of the isopropylidene group followed by the esterification of 6-OH-Glcp with 4-oxo-8-nonenoic acid gave diene **47** as the RCM precursor. Treatment of diene **47** with the Hoveyda–Grubbs catalyst and the subsequent hydrogenation over Wilkinson’s catalyst gave **48**. After the introduction of the cinnamate moiety and the removal of the TBS groups, ipomoeassin F (**6**) was finally obtained. In this route, ipomoeassin F was synthesized in 3.8% overall yield over 17 steps of the longest linear sequence from commercially available starting materials, and thus the synthesis can be easily scaled up to gram-scale (Zong et al., 2015).

**FIGURE 6:**
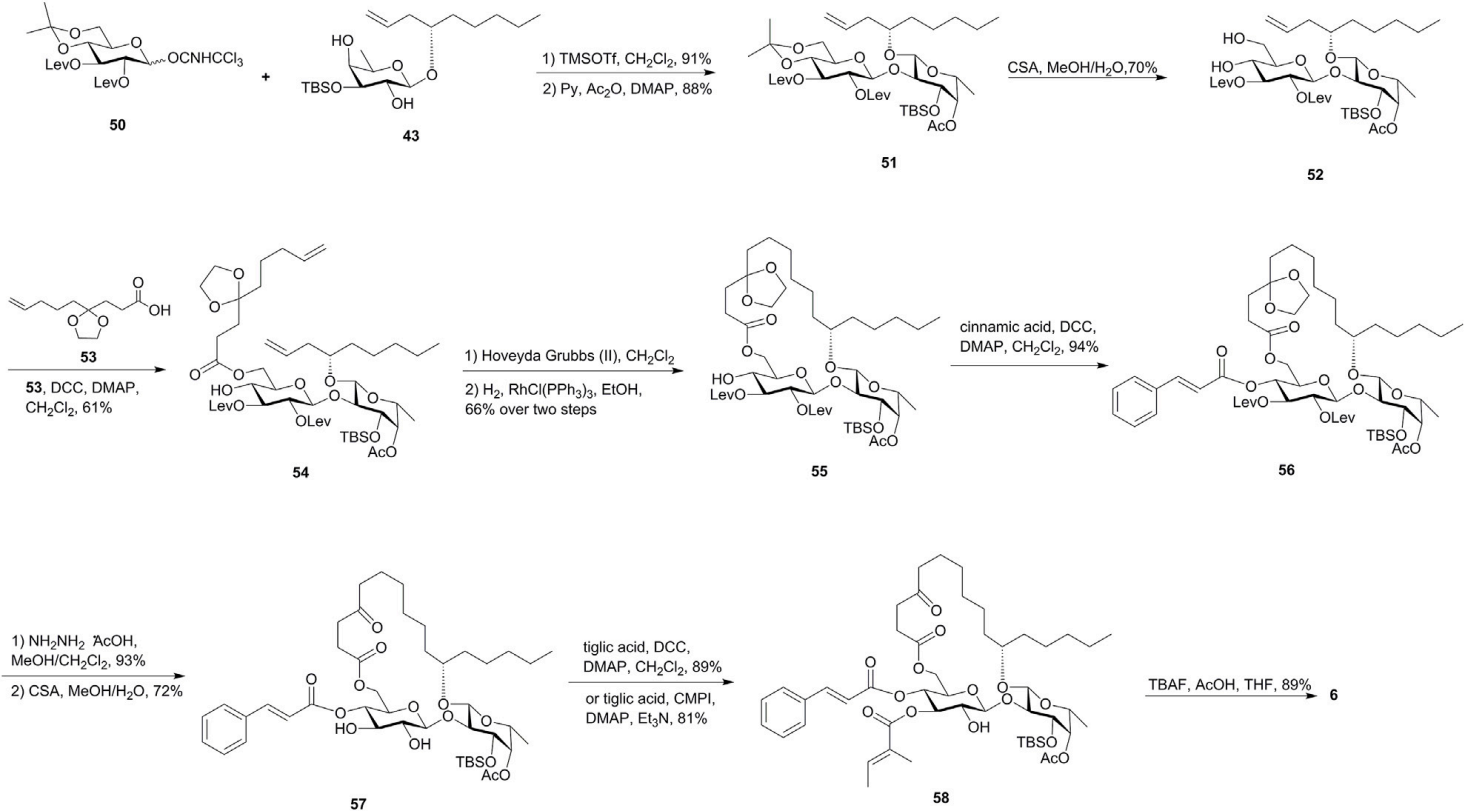
Second Shi’s approach toward the synthesis of ipomoeassin F (**6**). Lev, levulinoyl; CSA, camphor-10-sulfonic acid; CMPI, 2-chloro-1-methylpyridinium iodide.

In the second strategy, Shi’s group tried to install two acyl groups in the late stage by utilizing the highly regioselective esterification of the glucose diol (Figure 6). The disaccharide diol **52** was obtained by the removal of the isopropylidene group of **51**, which was built through regioselective glycosylation of the glucosyl donor **50** and fucoside acceptor **43**, followed by acetylation of the remaining 4-OH-Glcp. The chemoselective esterification of 6-OH-Glcp in diol **52** with carboxylic acid **53** gave diene **54** as the RCM precursor. The treatment of diene **54** with the Hoveyda–Grubbs catalyst and the subsequent hydrogenation reaction gave the ring structure **55**. The cinnamate moiety was then introduced using Steglich esterification to furnish disaccharide **56**. The removal of Lev groups followed by the cleavage of ketal in the presence of CSA gave the key intermediate **57**. Steglich or Mukaiyama esterification of **57** with tiglic acid yielded the desired 3-*O*-tigloyl product **58**, the TBS group of which was removed to afford ipomoeassin F (**6**) (Zong et al., 2017b). This route was developed with an overall yield of 4.0% over 17 steps of the longest linear sequence from commercially available D-glucose penta-acetate and was particularly suitable for the efficient preparation of ipomoeassin F analogues with modifications at 3-OH-Glcp.

Using these two strategies, Shi’s group also synthesized a series of ipomoeassin F analogues, including the 11*R*-epimer (**59**), acyl group-removed or -modified analogues (**30**, **60**–**71**), chain-modified or open-chain analogues (**72**–**83**), and monosaccharide analogues (**84**–**86**) (Zong et al., 2015; Zong et al. 2016; Zong et al. 2017a; Zong et al. 2017b; Zong et al. 2018; Zong et al. 2020, Figure 7). These analogues were evaluated for the cytotoxic activity, followed by the structure–activity relationship study. Based on the evaluation result, two α, β-unsaturated esters, the natural 11*S* configuration of aglycone, the size and flexibility of the ring, and the 6-membered ring of the fucoside moiety are critical for the activity, whereas, the cyclic skeleton, ketone in the aglycone, and 3-OH of the Fuc unit showed less influences on the activities of ipomoeassin F (**6**).

**FIGURE 7:**
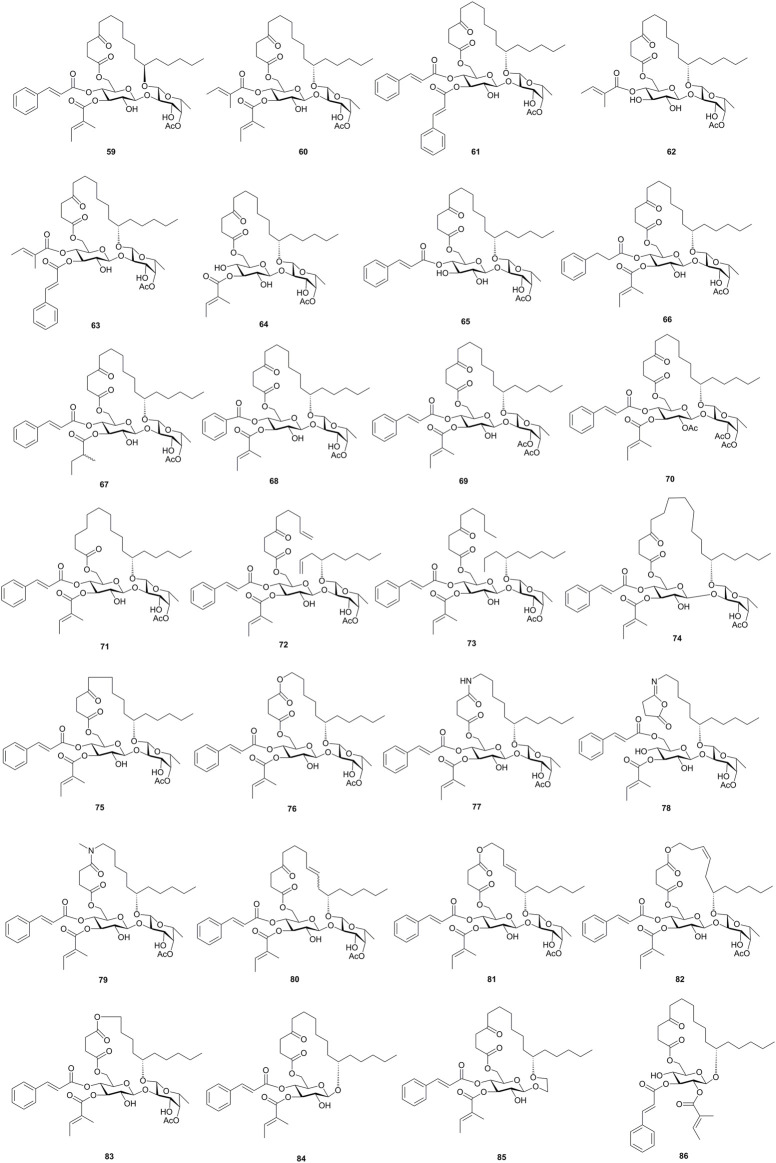
Structures of synthesized ipomoeassins F analogues **59**–**86**.

In addition, the biotin analogue of ipomoeassin F was synthesized by Shi’s group and was used to identify Sec61α, the pore-forming subunit of the Sec61protein translocon, as a direct binding partner of ipomoeassin F (**6**) (Zong et al., 2019). A ring-expanded ipomoeassin F analogue (**74**, Figure 7) exhibited improved cytotoxicity and *in vitro* protein translocation inhibition, implying that the binding pocket of Sec61α can accommodate further structural modifications, likely in the fatty acid portion (Zong et al., 2020). Due to the Sec61 inhibitory potential, ipomoeassin F (**6**) was developed as a valuable tool to research the protein biogenesis at the endoplasmic reticulum (Leznicki et al., 2022; O'Keefe et al., 2021a; 2021b; Roboti et al., 2021).

## Total synthesis of batatoside L (7)

### Approach by Yang’s group

Batatoside L (**7**) is a cytotoxic resin glycoside isolated from *Ipomoea batatas*, a plant with the common name of sweet potato (Yin et al., 2009). Due to the appealing structural and bioactive features, it was synthesized by Yang’s group. The crucial step was the construction of the 18-membered macrolactone framework through the Corey–Nicolaou macrolactonization approach (Xie et al., 2010, Figure 8).

**FIGURE 8:**
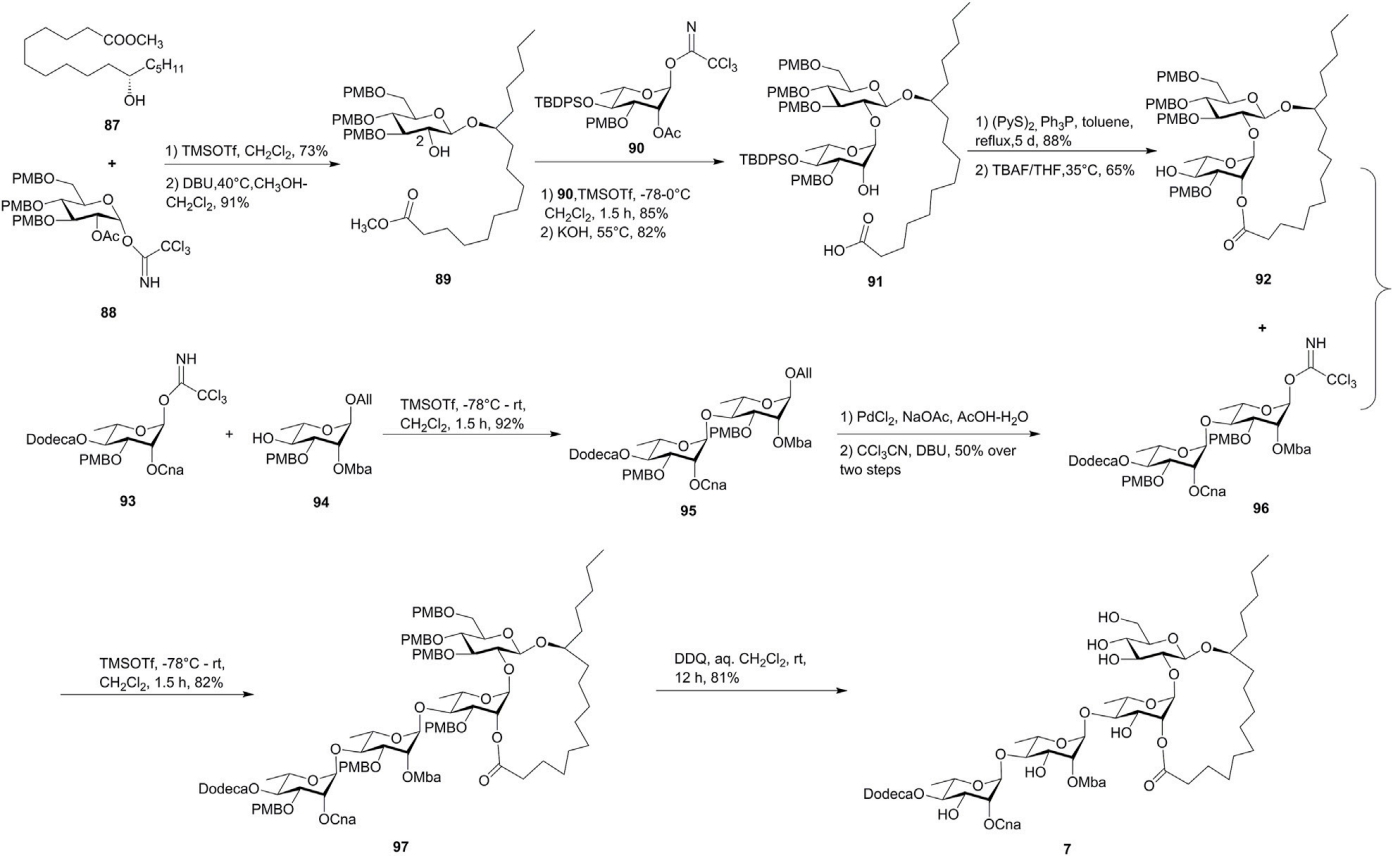
Yang’s approach toward the synthesis of batatoside L (**7**). Dodeca, n-dodecanoyl; Cna, *trans*-cinnamyl; Mba, 2*S*-methylbutyryl; All, allyl.

The synthetic approach started with the utilization of the synthesized chiral methyl 11(*S*)-jalapinolate (**87**) using commercially available (*R*)-glycidol as a starting material. The chiral hydroxyl ester (**87**) was glycosylated with D-glucosyl donors **88**, followed by the selective cleavage of C-2 ester functionality, to deliver *β*-D-glucopyranoside **89**. The subsequent coupling of **89** and *R*-L-rhamnopyranosyl trichloroacetimidate **90**, followed by the saponification with KOH, afforded the disaccharide acid **91**. The Corey–Nicolaou method was used for the macrolactonization, which yielded the desired lactone alcohol **92** by the removal of the silyl moiety. The exocyclic dirhamnopyranose fragment **95** was prepared by the glycosylation of monosaccharide modules **93** and **94** and was transformed to the trichloroacetimidate derivative **96**. Finally, the “inverse glycosylation” procedure developed by Schmidt et al. was used to form the tetrasaccharide **97** in 82% yield, which produced target batatoside L (**7**) by the cleavage of PMB-ether functions. The synthetic batatoside L (**7**) was found to be identical with the natural isolate on the basis of ^1^H and ^13^C NMR spectra and specific rotation comparisons (Xie et al., 2010).

## Total synthesis of murucoidins IV (8) and V (9)

### Approach by Wan’s group

Murucoidins IV (**8**) and V (**9**) were isolated from the flowers of *Ipomoea murucoides*, a plant with a vernacular name of “cazahuate” in Mexico (Chérigo and Pereda-Miranda. 2006). They present very similar structures, containing the same oligosaccharide chain and an 11*S*-hydroxyhexadecanoic acid aglycone. Their only difference lies in the macrolactone rings, which is an 18-membered ring and a 19-membered ring in murucoidins IV (**8**) and V (**9**), respectively. Different from the classic glycosylation reactions utilizing the trichloroacetimidate glycosyl donors or thioglycosyl donors, the interrupted Pummerer reaction-mediated (IPRm) glycosylation reactions were developed by Wan’s group and were used for the total synthesis of murucoidins IV (**8**) and V (**9**) (Fang et al., 2019, Figure 9).

**FIGURE 9:**
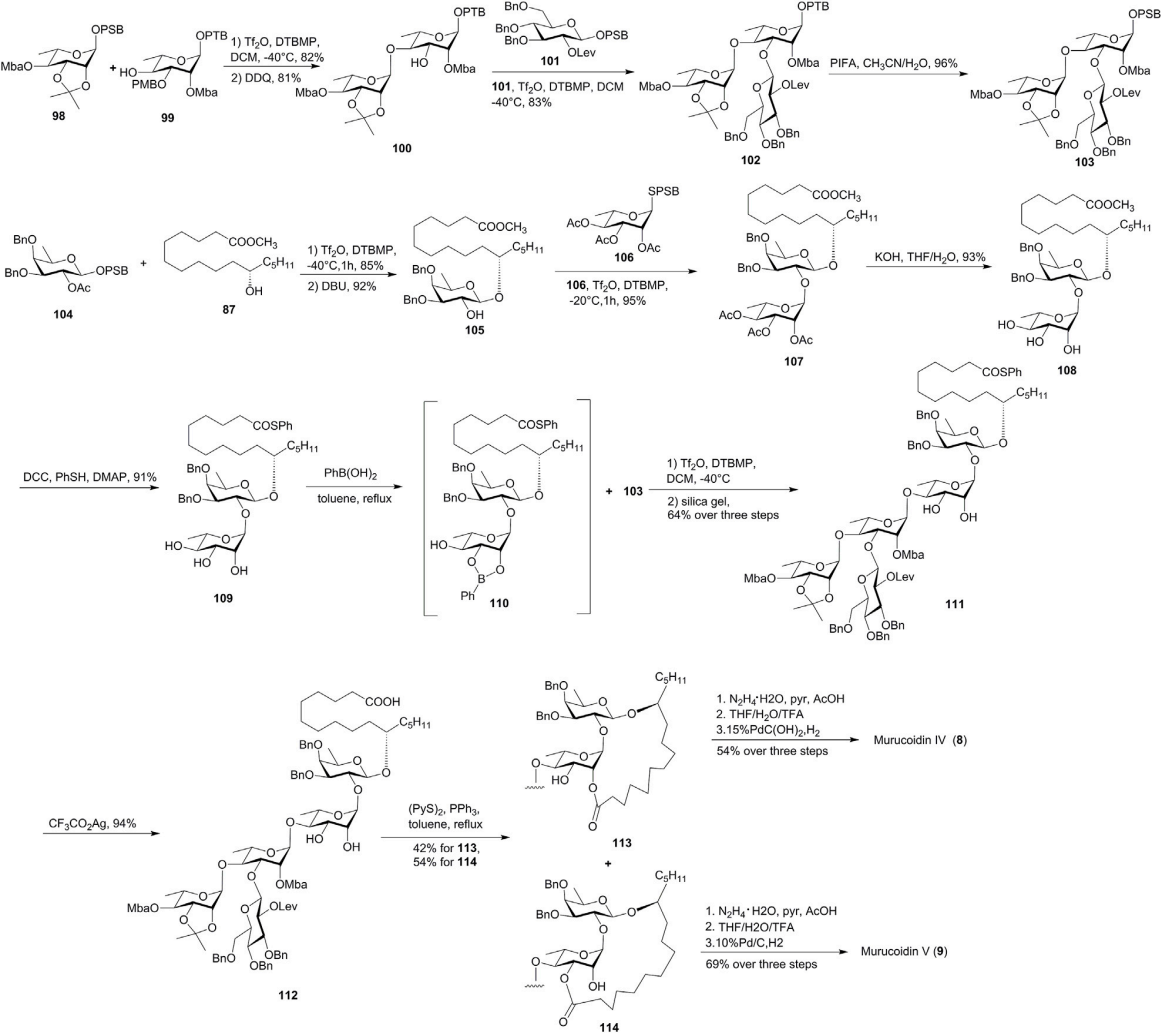
Wan’s approach toward the synthesis of murucoidins IV (**8**) and V (**9**). Mba, 2*S*-methylbutyryl; OPSB: *O*-2-[(propan-2-yl)sulfinyl]benzyl; OPTB, *O*-2-[(propan-2-yl)thio]benzyl; Lev, levulinoyl; PIFA, bis(trifluoro-acetoxyiodo)benzene; SPSB, *S*-2-[(propan-2-yl)sulfinyl] benzyl.

The coupling of latent OPTB glycoside **99** with active OPSB glycosyl donor **98**, followed by the removal of the PMB-protecting group, furnished latent disaccharide **100**. The glycosylation of **100** with OPSB glycoside **101** produced trisaccharide **102**, which was subsequently oxidized to yield exocyclic glycosyl donor **103** with the active OPSB group.

The synthesis of glycosyl acceptor fragment **108** was started with the glycosylation of glycosyl donor **104** with methyl 11(*S*)-jalapinolate (**87**). Release of the C2-hydroxyl group of the product gave acceptor **105**, which was coupled with the disarmed SPSB glycosyl donor **106** to provide disaccharide **107**. The global saponification of all the ester groups under basic conditions furnished the key intermediate **108**. Thioester was introduced for the protection of fatty acid to form **109**, and *cis*-dihydroxyl groups were protected by forming a cyclic boronic acid ester **110**. The site-selective [3 + 2] glycosidation of **110** and **103**, followed by the cleavage of the cyclic boronic ester using silica gel column chromatography, afforded pentasaccharide **111** in 64% total yield over three steps. The macrolactonization of **112** under Corey–Nicolaou conditions proceeded smoothly to furnish macrolactones **113** and **114** with 18- and 19-membered rings. Although there was no selectivity obtained for this macrolactonization, both skeletons belong to murucoidins IV (**8**) and V (**9**), respectively. Finally, global removal of the protecting groups of **113** and **114** accomplished the divergent synthesis of murucoidins IV (**8**) and V (**9**), respectively. The NMR data exactly matched with the natural isolate, which endorsed their real structures of murucoidins IV (**8**) and V (**9**) (Fang et al., 2019).

## Total synthesis of merremoside D (10)

Merremoside resin glycosides were isolated from *Merremia mammosa* (Bidara upas) and exhibited ionophoretic activity (Kitagawa et al., 1996a; Kitagawa et al., 1996b). Among them, merremoside D (**10**) was synthesized by two different strategies (Sharif et al., 2014; Xiao et al., 2020).

### Approach by O’Doherty’s group

A *de novo* asymmetric synthesis of merremoside D (**10**) based on pyranone glycosyl donor **115** and Corey–Nicolaou macrolactonization was proposed by O’Doherty and the co-workers (Sharif et al., 2014, Figure 10).

**FIGURE 10:**
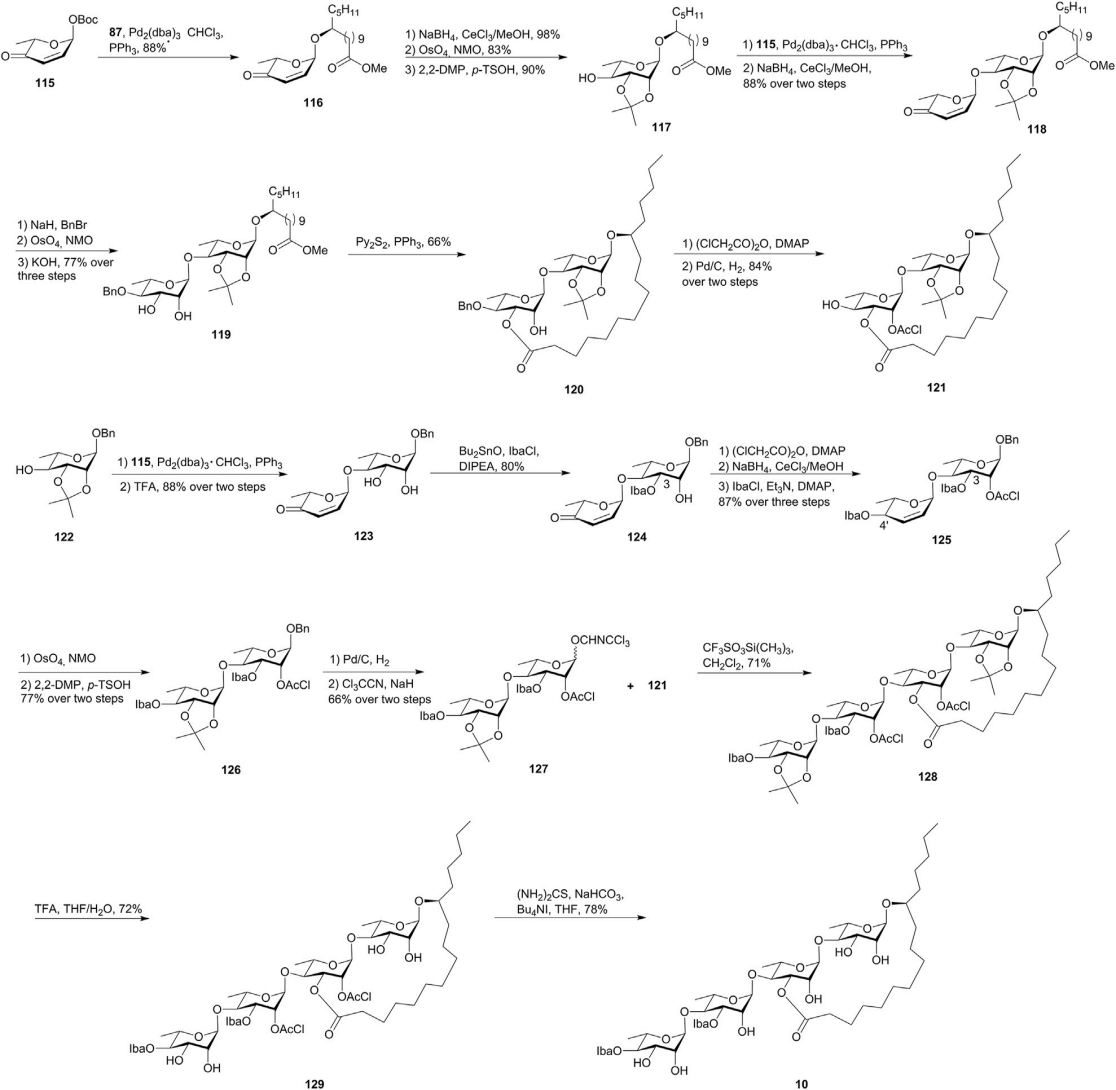
O’Doherty’s approach toward the synthesis of merremoside D (**10**). Iba, isobutyryl; DIPEA, N, N-diisopropylethylamine.

The synthesis of the macrolactone disaccharide began with a Pd-catalyzed stereoselective glycosylation of **115** with methyl 11(*S*)-jalapinolate (**87**) giving the enone **116**, which was converted to the glycosyl acceptor **117** *via* the Luche reduction, the Upjohn dihydroxylation and syn-diol protection. Then, a second Pd-catalyzed glycosylation of **117** with pyranone **115**, followed by enone reduction, gave allylic alcohol **118**. The benzylation of allylic alcohol **118**, followed by alkene dihydroxylation and methyl ester saponification, yielded diol-acid **119**. The macrolactonization of **119** under Corey–Nicolaou conditions gave macrolactone **120** as the major product (66% yield). The remaining hydroxyl group on macrolactone **120** was protected as chloroacetic ester, and benzylether was removed to form the glycosyl acceptor **121**.

The donor disaccharide was also prepared *via* the *de novo* asymmetric strategy. The protected rhamnopyranoside **122** was converted from pyranone **115** and then was linked by **115** *via* Pd-catalyzed glycosylation followed by acetonide removal to afford **123**. The introduction of the C-3 Iba-ester was accomplished *via* a tin-mediated esterification with isobutyryl chloride (IbaCl), and the C-2 hydroxyl group was then protected as chloroacetic ester, followed by the Luche reduction/esterification to yield allylic ester **125**. The glycosyl donor **127** was synthesized from **125** by functionalizing the double bond and anomeric activation and then reacted with the glycosyl acceptor **121** under Schmidt’s glycosylation conditions to afford **128** in 71% yield. Finally, the removal of acetonide groups and chloroacetates provided merremoside D (**10**). The comparison of the assignments of the synthetic product with the limited NMR data reported for the natural isolate allowed for the tentative confirmation of the structure of merremoside D (**10**). The total synthesis of merremoside D was achieved in 22 longest linear steps with a 3% overall yield and demonstrated the power of a *de novo* asymmetric approach to a stereochemically complex oligosaccharide natural product.

### Approach by Wan’s group

A novel one-pot relay glycosylation was established by Wan’s group and was used for the synthesis of merremoside D (**10**). The method capitalizes on the *in situ* generated cyclic-thiosulfonium ion as the relay activator, which directly activates the newly formed thioglycoside in one pot and thus constructs two glycosidic bonds with only one equivalent of triflic anhydride (Xiao et al., 2020, Figure 11).

**FIGURE 11:**
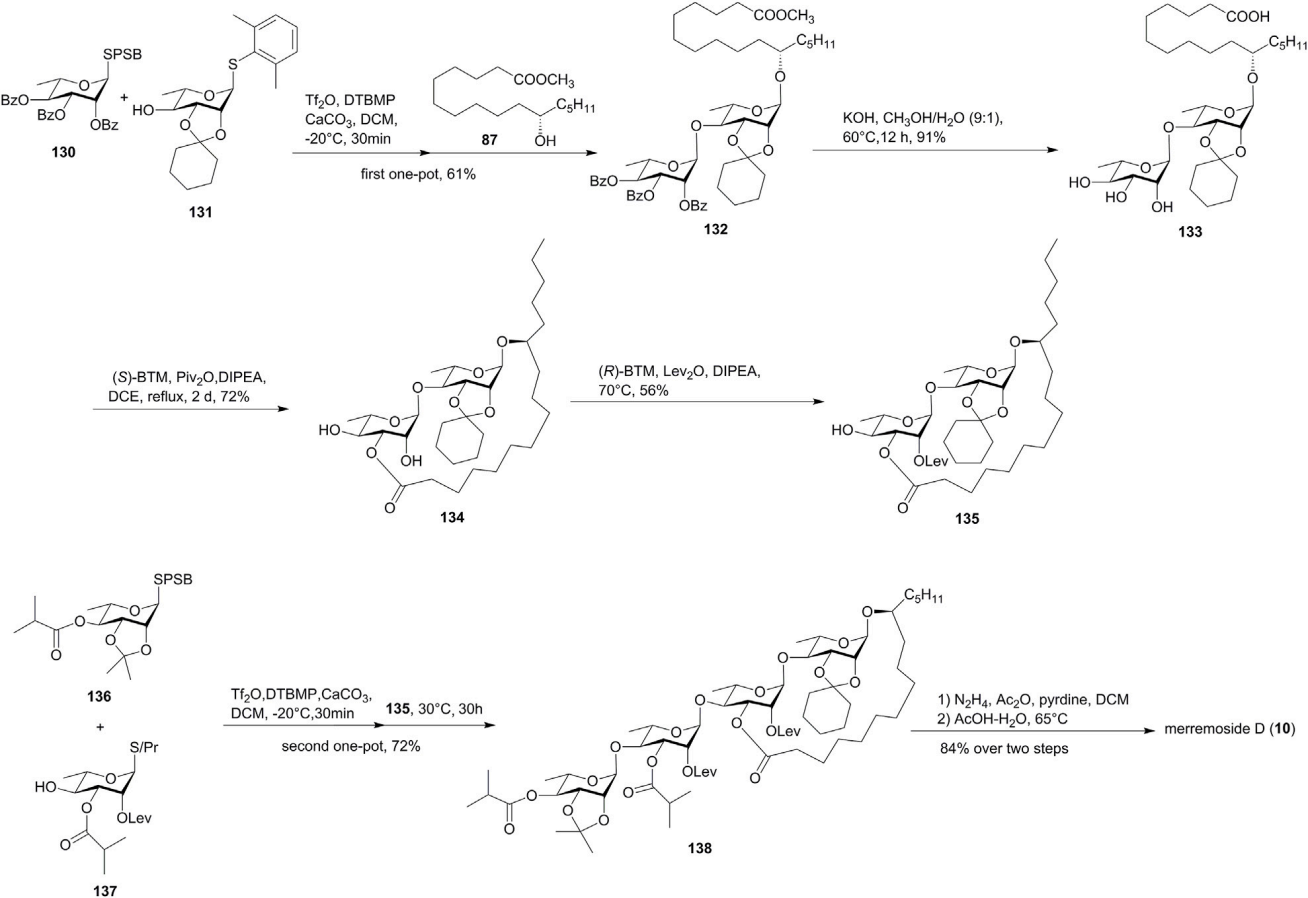
Wan’s approach toward the synthesis of merremoside D (**10**). SPSB, *S*-2-[(propan-2-yl)sulfinyl] benzyl; BTM, benzotetramisole; DIPEA, N, N-diisopropylethylamine; Lev: levulinoyl.

Merremoside D (**10**) was synthesized with two key one-pot relay glycosylations. The first one-pot reaction produced disaccharide **132** by the coupling of SPSB glycoside **130**, thioglycoside **131**, and methyl 11(*S*)-jalapinolate (**87**). The hydrolyzation of the ester groups in **132** afforded acid **133** with three continuous free hydroxy groups. The cation–n interaction-mediated site-selective acylation reaction was used for the construction of the macrolide structure. The site-selective macrolactonization of **133** with 1.5 equiv of (*S*)-BTM (benzotetramisole) along with 1.0 equiv of Piv_2_O as esterification reagents gave **134** in 72% yield. The following site-selective installation of the levulinoyl (Lev) group on the C2′ position produced **135**. The second one-pot glycosylation of **135** with monosaccharide modules **136** and **137** produced the desired tetrasccharide **138** in 72% yield, which eventually furnished merremoside D (**10**) by global deprotection.

## Total synthesis of tricolorin A (11)

### Approach by Wan’s group

Tricolorin A (**11**) is a resin glycoside isolated from *Ipomoea tricolor* (heavenly blue morning glory) (Pereda-Miranda et al., 1993; Bah and Pereda-Miranda. 1996). The structure of tricolorin A (**11**) consists of a 19-membered macrolactone and a tetrasaccharide backbone. Tricolorin A (**11**) had been synthesized by several groups before 2009 (Pereda-Miranda et al., 2010). Herein, we introduced the recently reported synthetic approach by Wan’s group *via* the IPRm glycosylation and one-pot relay glycosylation (Sun et al., 2020, Figure 12).

**FIGURE 12:**
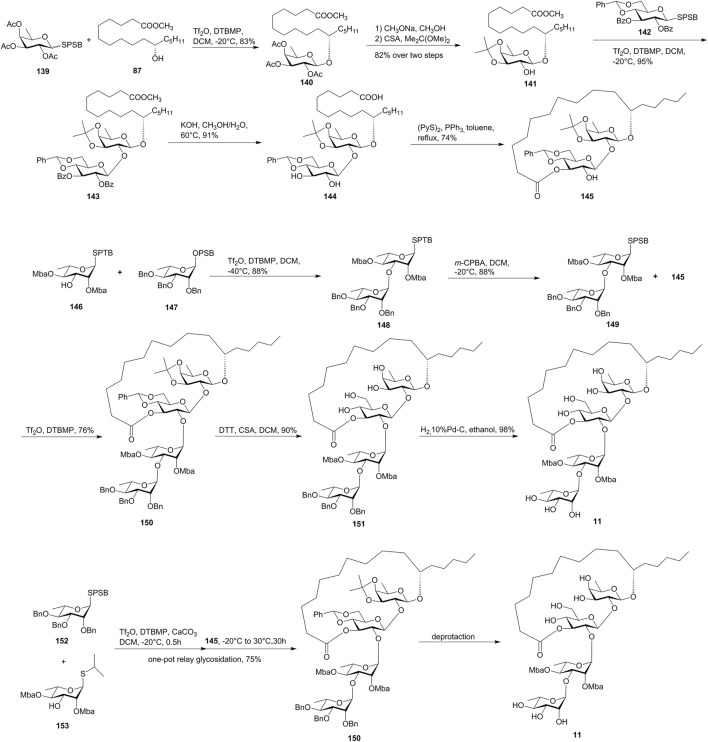
Wan’s approaches toward the synthesis of tricolorin A (**11**). SPSB, *S*-2-[(propan-2-yl)sulfinyl]benzyl; CSA, camphor-10-sulfonic acid; Mba, 2*S*-methylbutyryl; SPTB: *S*-2-[(propan-2-yl)sulfanyl]benzyl; OPSB, *O*-2-[(propan-2-yl) sulfinyl]benzyl.

The macrolactone fragment was prepared using IPRm glycosylation, which was started with the glycosylation of active SPSB donor **139** with methyl 11(*S*)-jalapinolate (**87**). The subsequent deacetylation and isopropylidenization of **140** afforded glycosyl acceptor **141**, which was coupled with the SPSB donor **142** under the IPRm glycosylation conditions to furnish disaccharide **143**. The saponification of **143** released the free dihydroxy groups and the aglycon carboxyl group, which formed the desired 19-membered macrolactone **145** under the Corey–Nicolaou condition in 74% yield.

The installation of the donor disaccharide fragment could be both achieved by IPRm glycosylation or one-pot relay glycosylation.

In the IPRm glycosylation approach, coupling of SPTB glycosyl acceptor **146** with the OPSB glycosyl donor **147** under IPRm glycosylation conditions produced disaccharide **148**, which was then selectively oxidized to the SPSB donor **149**. Coupling of the two key building blocks **149** and **145** under the standard IPRm glycosylation conditions furnished the desired tetrasaccharide **150** in moderate 1,2-trans selectivity (76% yield), while using the corresponding OPSB donor of **149** produced **150** in 92% yield. The removal of isopropylidene and benzylidene acetal groups of tetrasaccharide **150**, followed by the final debenzylation, yielded tricolorin A (**11**) (Figure 12). The NMR data exactly matched with the natural and the reported synthetic tricolorin A (**11**). The longest linear sequence in this route is 16 steps with 12.5% overall yield.

In the one-pot relay glycosylation approach, monosaccharide unit **152** was first coupled with **153** in the presence of Tf_2_O, DTBMP, and CaCO_3_, and then macrolactone **145** was added to yield precursor tetrasaccharide **150** in 75% yield in one-pot glycosylation (Figure 12). Compared to the stepwise synthesis, the one-pot procedure offered a slightly higher overall yield and great convenience.

## Total synthesis of tricolorin F (12)

### Approach by Sakairi’s group

Tricolorin F (**12**) is another resin glycoside isolated from *I. tricolor*. It is a linear hetero-trisaccharide of jalapinolic acid, with the macrolactone bond linked at the C-2 position to the third sugar unit to form a 21-membered ring (Bah and Pereda-Miranda. 1996).

Sakairi’s group provided an intramolecular glycosylation for the construction of the macrolide structures of resin glycosides, which was applied for the total synthesis of tricolorin F (**12**) (Son et al., 2009, Figure 13). The D-fucosyl donor **154** was first coupled with aglycone methyl 11(*S*)-jalapinolate (**87**), followed by Zemplén de-O-benzoylation, giving the D-fucosyl acceptor **141**. The glycosylation of **141** with the donor **155** under the MeOTf-promoted glycosylation, followed by hydrolyzation with aqueous NaOH, afforded the disaccharide **156**. The coupling of **156** with excess thioquinovoside **157** gave the trisaccharide **158**. Then, the intramolecular glycosylation of **158** using the MeOTf method gave the fully protected derivative **159** in 70% yield, which afforded tricolorin F (**12**) by global deprotection. In addition to macrolactonization and RCM, this new methodology would provide various synthetic analogues useful for the systematic survey of the biological functions of resin glycosides.

**FIGURE 13:**
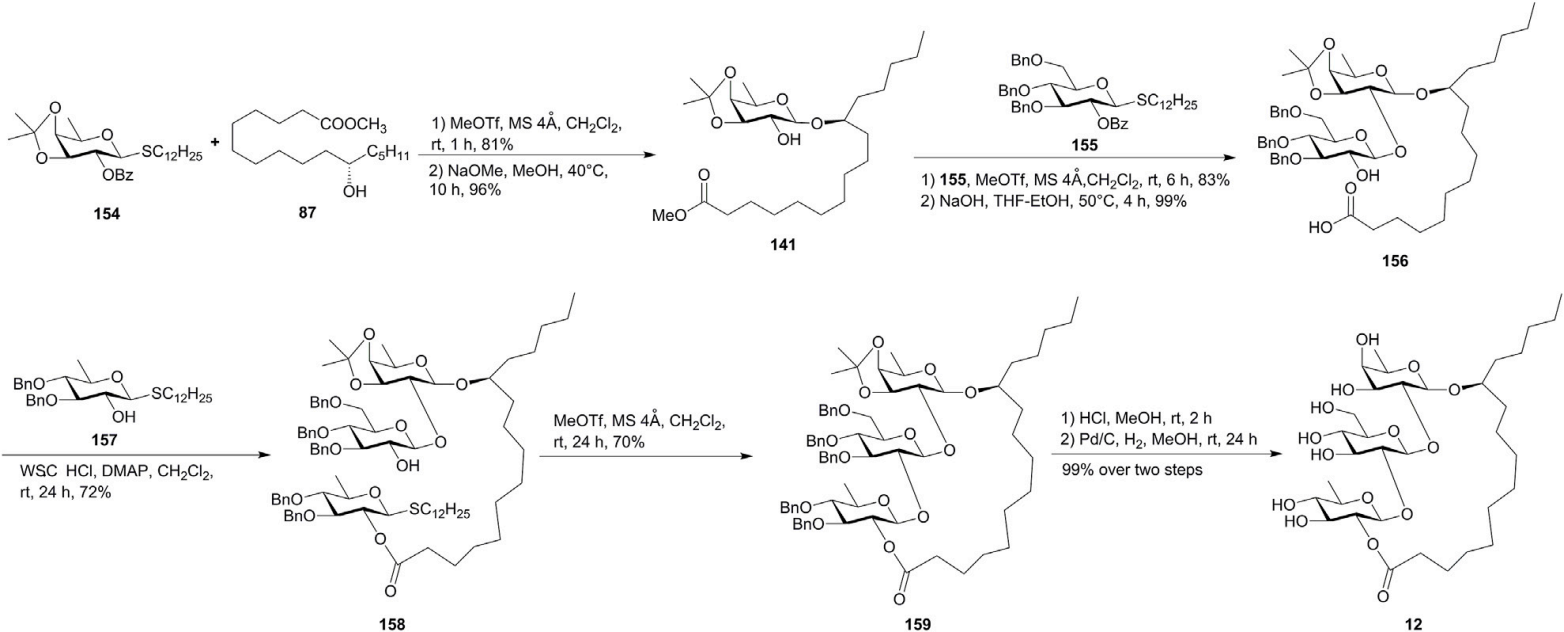
Sakairi’s approach toward the synthesis of tricolorin F (**12**). WSCHCl, 1-(3-dimethylaminopropyl)-3-ethylcarbodiimide hydrochloride.

## Total synthesis of batatin VI (13)

### Approach by Yang’s group

Batatin VI (**13**) is an ester-type resin glycoside dimer isolated from *Ipomoea batatas* (Rosas-Ramírez et al., 2011) and was synthesized by Yang’s group *via* a convergent [5 + 3] glycosidic coupling approach. An effective Keck macrolactonization was utilized for the formation of the 18-membered ring (Zhu et al., 2013, Figure 14).

**FIGURE 14:**
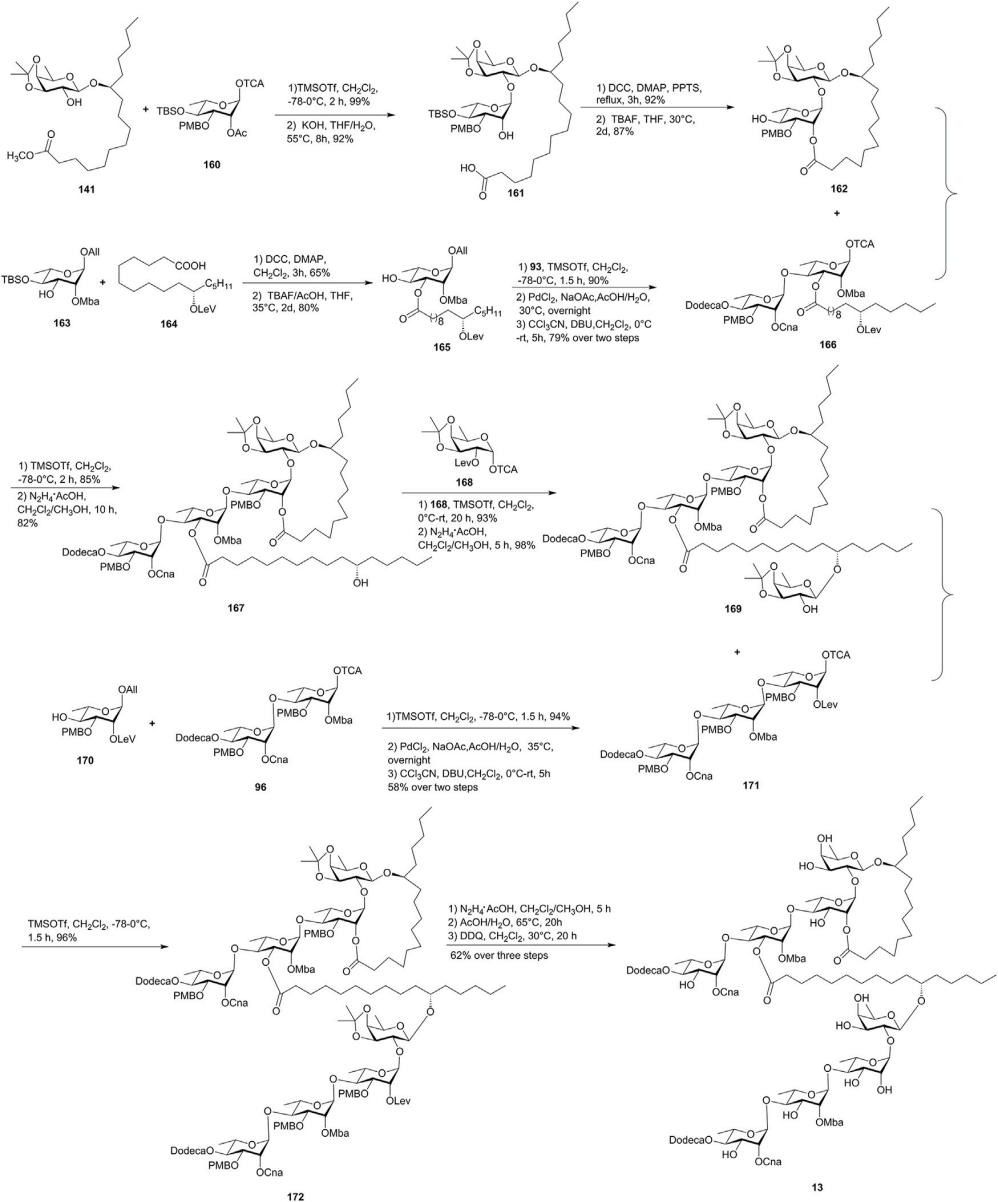
Yang’s approach toward the synthesis of batatin VI (**13**). All, allyl; Mba, 2*S*-methylbutyryl; Lev, levulinoyl; Dodeca, n-dodecanoyl; Cna, *trans*-cinnamyl.

The macrolactone precursor **161** was obtained by the Schmidt glycosylation of the known **141** with monosaccharide **160**, followed by the saponification with KOH. Then, the Keck macrolactonization followed by the subsequent desilylation gave the required lactone alcohol **162** in 87% yield. The disaccharide donor **166** was obtained by the coupling of **165** with the chiral rhamnosyl intermediate **93**, followed by the deallylation and activation of the resulting crude hemiacetal. The macrocycle acceptor **162** was coupled with **166** under TMSOTf-activated inverse glycosylation conditions in 85% yield, and then the levulinate was selectively hydrolyzed to afford tetrasaccharide **167**. The glycosylation of **167** with monosaccharide **168** and the removal of the Lev group afforded crucial the pentasaccharide intermediate **169**. Meanwhile, the trisaccharide intermediate **171** was obtained by the glycosidic coupling of **170** with **96**. Finally, the fragments **169** and **171** were also linked together under TMSOTf-activated inverse glycosylation conditions to furnish protected octasaccharide **172** in 96% yield. The following global deprotection of **172** afforded the target batatin VI (**13**). Unfortunately, the NMR data of the synthetic product were not in full agreement with those of the naturally occurring batatin VI (**13**), suggesting that a structural revision to batatin VI (**13**) may be required (Zhu et al., 2013).

## Synthesis of resin glycoside intermediates

**FIGURE 15:**
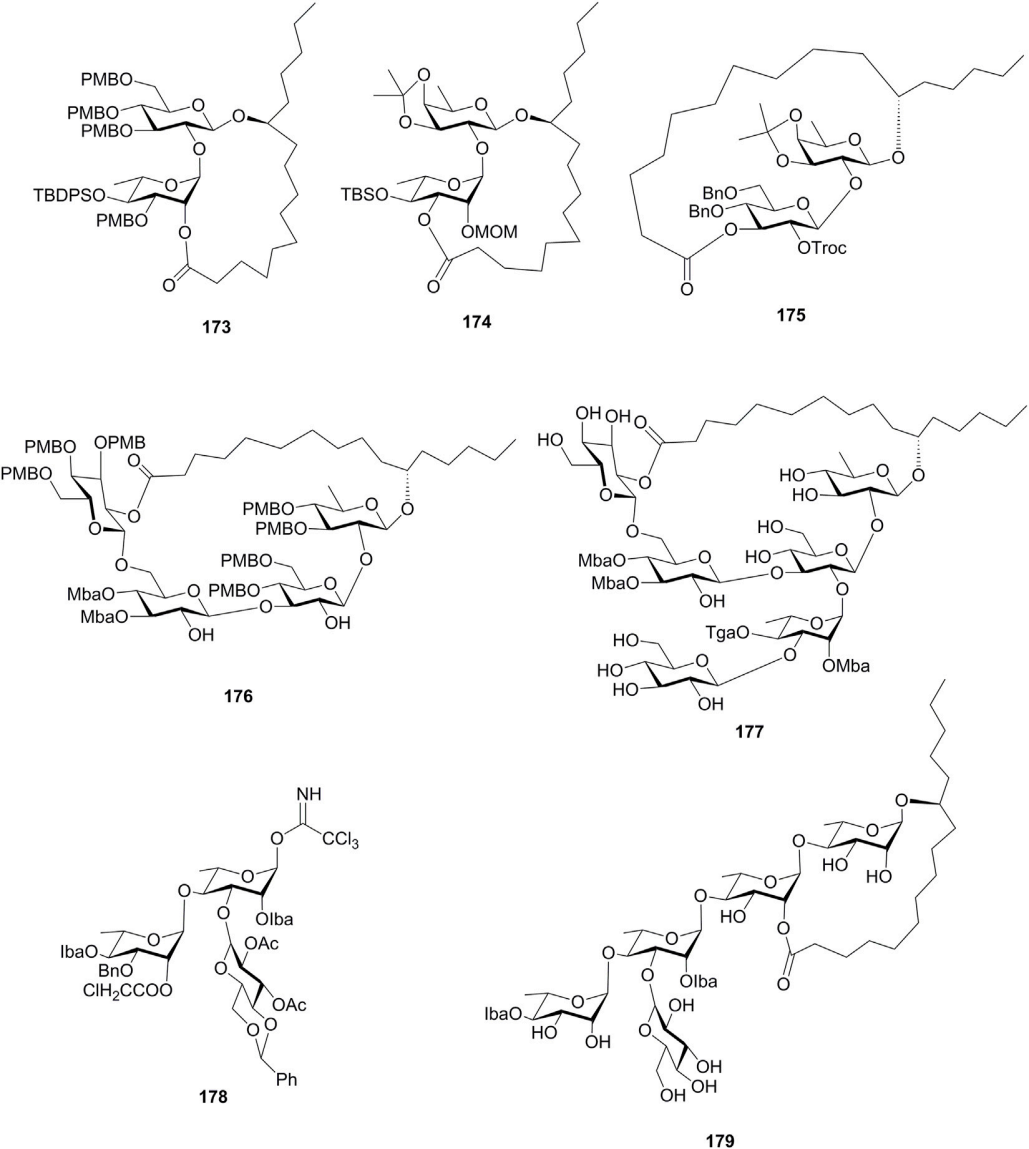
Structures of **173**–**179**. TBDPS, *tert*-butyldiphenylsilyl; Troc, trichloroethoxycarbonyl; Mba, 2*S*-methylbutyryl; Tga, tiglyl; Iba, isobutyryl.

There are also some research studies on the efficient synthesis of resin glycoside intermediates, which are summarized in Figure 15 and were introduced here briefly. Zhang’s group tried the Keck macrolactonization method to synthesize the macrolactone structures **159**, **173**, and **174** (Huang et al., 2015). The intramolecular glycosylation method was used for the construction of the macrolide cores **175** and **176**, which were the intermediates for tricolorin A (**11**) and calysolin IX (**177**) (Son et al., 2009; Nawój et al., 2020). In addition, a suitably protected trichloroacetimidate (**178**) was synthesized by a block synthesis approach, which could serve as an exocyclic intermediate in the total synthesis of resin glycoside merremoside H_2_ (**179**) (Shen et al., 2009).

## Conclusion

Given the structural diversification and promising bioactivities, considerable progress has been made in the total synthesis of resin glycosides. In this review, we highlighted reports on various total syntheses of resin glycosides in the last decade.

The construction of the macrolide structure is the most crucial step. The ring-closing metathesis (RCM) method was the major approach for the synthesis of resin glycoside ipomoeassins A–F (**1**–**6**), especially Shi’s group developed two strategies for the synthesis of ipomoeassin F (**6**) and its analogues, which were used for comprehensive structure–activity relationship studies on the cytotoxic activity. Several macrolactonization approaches were also used for the construction of the macrolide structure, including the Corey–Nicolaou macrolactonization reaction for batatoside L (**7**), murucoidins IV (**8**) and V (**9**), merremoside D (**10**), and tricolorin A (**11**), the cation–n interaction-mediated site-selective acylation reaction for merremoside D (**10**), and the Keck macrolactonization reaction for batatin VI (**13**) and the macrolactone intermediates **159**, **173**, and **174**. In addition, the intramolecular glycosylation method was used for the total synthesis of tricolorin F (**12**), as well as the macrolide cores **175** and **176**.

The construction of the glycosidic bond is another important step. Although the common activation of trichloroacetimidate glycosyl donors or thioglycosyl donors were the mostly used glycosylation methods, Wan’s group have developed the interrupted Pummerer reaction-mediated (IPRm) glycosylation and one-pot relay glycosylation methods, which were successfully utilized for the synthesis of murucoidins IV (**8**) and V (**9**), merremoside D (**10**), and tricolorin A (**11**). A Pd-catalyzed stereoselective glycosylation was used for the synthesis of merremoside D (**10**).

In general, the synthesized resin glycosides and their analogues helped in structural verification, structural modification, and further biological activity exploration, as well as provided enlightenment for the synthesis of glycoside compounds. However, compared with the vigorous structure and bioactivity studies, only a limited number of bioactive resin glycosides have been synthesized. The development of novel methods for the synthesis of resin glycosides and their analogues with better biological outcomes would be of strategic importance for further drug discovery.
